# Exploring surface charge dynamics: implications for AFM height measurements in 2D materials

**DOI:** 10.3762/bjnano.15.64

**Published:** 2024-07-01

**Authors:** Mario Navarro-Rodriguez, Andres M Somoza, Elisa Palacios-Lidon

**Affiliations:** 1 Centro de Investigación en Óptica y Nanofísica (CIOyN), Department of Physics, University of Murcia, E-30100, Spain

**Keywords:** 2D materials, incorrect height measurements, Joule dissipation, surface conductivity, tip influence

## Abstract

An often observed artifact in atomic force microscopy investigations of individual monolayer flakes of 2D materials is the inaccurate height derived from topography images, often attributed to capillary or electrostatic forces. Here, we show the existence of a Joule dissipative mechanism related to charge dynamics and supplementing the dissipation due to capillary forces. This particular mechanism arises from the surface conductivity and assumes significance specially in the context of 2D materials on insulating supports. In such scenarios, the oscillating tip induces in-plane charge currents that in many circumstances constitute the main dissipative contribution to amplitude reduction and, consequently, affect the measured height. To investigate this phenomenon, we conduct measurements on monolayer flakes of co-deposited graphene oxide and reduced graphene oxide. Subsequently, we introduce a general model that elucidates our observations. This approach offers valuable insights into the dynamics of surface charges and their intricate interaction with the tip.

## Introduction

Two-dimensional (2D) materials have emerged as a promising platform for next-generation electronic devices [1], sensors [2], and biomedical applications [3–4], among other areas [5–8]. Graphene-related materials [8], transition metal dichalcogenides [9], boron nitride [10], and MXenes [11], among many others, exhibit novel and exotic properties, which markedly differ from their bulk counterparts [12]. This has sparked considerable interest spanning from fundamental research to practical device applications. The distinctive physical and chemical properties of 2D materials, composed of one atom- or a few atom-thick sheets, stem from their thin, flat structure, providing an exceptional surface-to-volume ratio. Moreover, their extensive surface exposure renders them highly sensitive to ambient and external influences, amplifying chemical reactivity [13] and bestowing attractive properties regarding electrochemical and catalytic reactions [14]. Furthermore, the ability to stack these materials facilitates the creation of new heterostructures with tailored properties [15–16], making 2D materials suitable for different applications.

Understanding the correlation between structural and topographical variations and their impact on mechanical [17–18], optical [19–20], magnetic [21–22], electronic [23–24], or electrochemical properties [25] is a key topic of research. Factors such as flake size and shape, composition, density of defects, or doping significantly influence the response of 2D materials. Given the nanoscopic scale underlying the functionality of 2D materials, atomic force microscopy (AFM) techniques emerge as ideal tools to investigate them [26–27]. Depending on the operation mode and under controlled environmental conditions, AFM offers the possibility to record morphology along with relevant electronic, mechanical, or magnetic properties with nanoscale resolution. In addition, it can be integrated with classical optical spectroscopy methods such as Raman and fluorescence [20,28–29], enabling a multidimensional characterization approach.

A well-recognized issue within the AFM community is the inaccurate height determination derived from topography images on heterogeneous samples. This discrepancy arises from various sources depending on the operation mode and working parameters. In the frequency modulation mode (FM-AFM), a non-compensated bias voltage between tip and sample, from differences in the surface potential (SP), results in inaccurate height measurements [30]. This issue can be addressed with Kelvin probe force microscopy (KPFM). Under ambient conditions, the most common mode is amplitude modulation (AM-AFM), which uses the oscillation amplitude reduction as the input for the topography feedback. Its main aspects are summarized in [31]. At large free oscillation amplitudes, the tip mechanically touches the surface during part of the oscillation. This mode is known as “intermittent contact” or tapping mode, and incorrect height measurements are usually ascribed to variations in the local elasticity [32–33] or differences in the local adhesion, related to differences of the wetting properties [34]. At moderate oscillation amplitudes, intimate tip–sample contact is avoided, and the energy dissipation takes place at the lower turning point of the oscillation cycle because of the formation and rupture of liquid necks [35–37]. When operating in this less invasive mode, the driving excitation frequency can be fixed at, or near, the free resonance frequency of the cantilever, or tracked by using a phase-locked loop (PLL) to keep the system always in resonance. If the driving excitation frequency is kept fixed, the phase variations contain information about the dissipation. In this mode, the amplitude reduction may be due to (i) the tip–sample interaction (conservative or non-conservative), which shifts the resonance frequency and, therefore, makes the excitation go out of resonance, (ii) non-conservative interactions, which dissipate parts of the system’s energy and, thus, reduce the amplitude, or (iii) a combination of both. Operating in this mode, erroneous height measurements derived from topography images are then attributed to local variations of hydrophilicity or hydrophobicity, which affect the dissipative capillary forces between tip and sample [38–41], or to typically electrostatic conservative forces [42]. In the latter case, using KPFM to minimize these forces mitigates the problem. Finally, if the driving excitation frequency is tracked to follow the resonance frequency shift induced by the tip–sample interactions, the system is always excited at resonance. Then, the amplitude is reduced because of non-conservative forces only, and topography images can be understood as constant-dissipation images [43]. Larger dissipative interactions necessitate the tip to retract further from the surface to maintain a constant amplitude. Consequently, in this mode, highly hydrophilic materials may appear thicker than their hydrophobic counterparts [39–40].

Erroneous height measurements are especially important in AFM studies of 2D materials [8,44–46]. The use of dynamic modes enhanced the problem; measured heights tend to be overestimated, especially in comparison to those obtained in contact mode [8,47–52]. As discussed above, the extent of the discrepancy depends on the operation mode [53–54] and environmental conditions [8] and is notably pronounced in samples grown on insulating substrates [55–57]. The origin of incorrect height measurements in 2D materials has been ascribed to the same sources as those mentioned above, including capillary forces and adhesion [8,46], electrostatic forces [58–60], or residues of the solvent [8]. However, in many cases, this is not enough to fully explain the measurements, suggesting that there may be additional interactions directly related to the 2D nature of these materials affecting the height measurements.

To explore this issue, in this work, we conducted a study on single-layer flakes of graphene oxide (GO) and reduced graphene oxide (rGO) co-deposited on an insulating substrate. Measurements on these two materials, which exhibit very different properties in terms of hydrophilicity and conductivity, allowed us to clarify the most relevant factors of the problem and how they affect the apparent height measured with AM-AFM. This has enabled us to identify, in addition to the previously described interactions, an additional contribution to the tip–sample interaction due to the movement of charges on the surface induced by the oscillating tip. To understand and quantify this mechanism, we have proposed a very general model that solves Maxwell’s equations for the system, including the presence of the tip, which we have subsequently particularized for 2D materials on insulating supports.

## Experimental

Co-deposited samples of GO and rGO were prepared following the methodology outlined in [61]. In summary, ultradiluted (4 × 10^−4^ wt %) dispersions of GO and/or rGO in Milli-Q type-I water (MQ water) were utilized. A drop of these dispersions was cast onto highly doped p-type silicon (1–10 Ω·cm, Siltronix) with a 300 nm SiO_2_ layer thermally grown on top. Before deposition, the substrate underwent a thorough cleaning process, which involved rinsing with ethanol and MQ water. Subsequently, the substrate was exposed to UV/ozone for 15 min to eliminate organic contaminants and promote the hydrophilicity of the SiO_2_ surface. GO (Graphenea), was employed without further treatment, while rGO was obtained through chemical reduction using hydrazine hydrate (50–60%, Sigma-Aldrich). After deposition, we heated the sample for a minimum of 3 h at 60 °C on a hot plate to remove some of the physisorbed water. While still hot, it was transferred to the AFM and left to cool down in a nitrogen atmosphere. Before starting measurements under controlled humidity, we waited for a minimum of 1 h until the humidity stabilized.

The experiments were performed at room temperature and low relative humidity (RH < 10%) in a dry nitrogen atmosphere. Topography images were acquired in AM-AFM mode by using the oscillation amplitude as the topography feedback channel. To maintain the system at resonance and track the frequency shift, a wide-bandwidth PLL (0.5–32 kHz) was enabled. In this operation mode, the amplitude signal carries information about dissipative interactions, as conservative forces only modify the resonant frequency. KPFM was operated in the frequency modulation mode (FM-KPFM) with an AC voltage of 700 mV at 7 kHz using platinum-coated silicon tips (Olympus AC240TM-R3, *k* = 2 N/m and *f*_0_ = 70 kHz). Using a dual lock-in amplifier (Zurich instruments, HF2LI), in addition to the KPFM channel, which provides information on the sample's SP, the 2ω_elec_ capacitance signal was also recorded, as explained in [61].

Spectroscopy data were acquired using a variant of the 3D-mode dynamic force spectroscopy [62], explained in detail in [63]. Briefly, force, frequency shift, amplitude, and phase channels are recorded simultaneously at a fixed sample point as a function of the applied bias voltage (*V*_bias_) for different tip–sample distances (*z*). This way, interaction images *I*(*V*_bias_, *z*) are obtained and later processed to obtain the relevant information from each channel.

## Results and Discussion

GO and rGO belong to the graphene family. In GO, the carbon basal plane is randomly decorated with oxygen-containing functional groups, including hydroxy, epoxy, and carboxyl groups [64–65]. In contrast, rGO is derived from the partial removal of these functionalities [66–69]. Further details about the structure and properties of GO and rGO are given in section SI.1 of Supporting Information File 1. Beyond their significance in various applications such as materials science, electronics, and biomedicine [70], these materials serve as benchmark systems for fundamental studies in 2D materials because of their markedly distinct properties [71]. Specifically, GO is a hydrophilic insulating material [72], while rGO exhibits a hydrophobic and more conductive nature [69,73–75], both dependent on the degree of reduction. This stark contrast provides an ideal heterogeneous sample to study the tip–sample interaction of co-deposited GO and rGO on insulating substrates.

**Figure 1:**
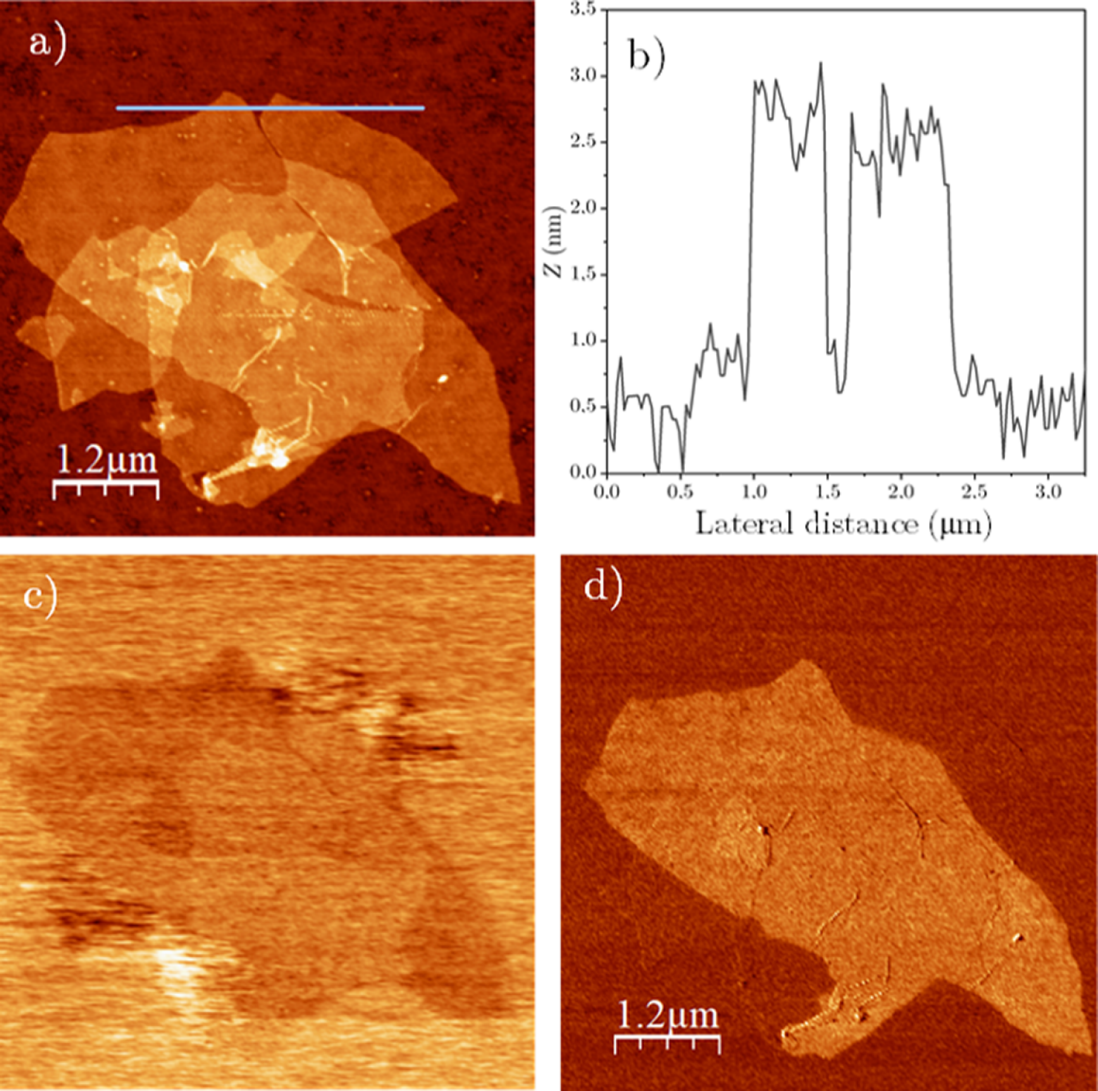
(a) Topography of a stack of GO and rGO flakes, *z* scale = 10 nm. (b) Profile along the blue line in (a). (c) KPFM (*z* scale = 320 mV) and (d) 2ω_elec_ images corresponding to the topography in (a).

In Figure 1, we present a stack of single-layer GO and rGO flakes co-deposited on SiO_2_, measured under N_2_ atmosphere (RH < 10%). Distinguishing between GO and rGO flakes based solely on the topography image proves to be challenging, as both materials exhibit similar heights. Therefore, additional AFM interaction channels are necessary for accurate differentiation. As explained in [61], this distinction is achieved unequivocally by using the KPFM and 2ω_elec_ electrostatic channels. In KPFM images (Figure 1c), GO flakes are identified by their signature localized charge domains related to its low conduction and disordered nature [76]. Conversely, rGO presents a roughly uniform surface potential consistent with the presence of larger sp^2^ regions, which increase the localization length, and with its enhanced conductivity as compared to GO. The 2ω_elec_ signal (Figure 1d), related to the tip–sample capacitance [77], also allows one to discriminate GO from rGO. While a quantitative interpretation of the 2ω_elec_ contrast in 2D materials necessitates further elucidation, it has proven reliable and robust for distinguishing materials with distinct electronic properties and, in particular, for identifying rGO with different reduction degrees [61]. In this channel, GO displays no contrast, whereas rGO shows a larger value compared with the substrate. With the unambiguous identification of each type of flake, we can proceed to study their thickness and its dependency on various external parameters. A line profile comprising both types of flake (Figure 1b) reveals a height of approximately 2 nm, larger than the expected nominal heights of ≈0.9 nm for GO and ≈0.3 nm for rGO. Moreover, the rGO flake appears to be slightly thicker than the GO flake, contradicting the anticipated reduction in thickness due to the removal of functionalities during the transition from GO to rGO. As mentioned above, in our AM-AFM measurements, the inconsistencies in height arise from the dissipative contrast between different materials. Under ambient conditions, this is typically dominated by capillary forces [39–40]. However, despite rGO being hydrophobic and both GO and the SiO_2_ substrate being hydrophilic, the rGO sheet is observed as thicker than the GO sheet, where the formation of liquid necks between the tip and the sample should be more favorable [37]. It is worth noting that the measurements in Figure 1 were conducted at RH < 10% to minimize any potential influence of this effect, although no significant differences were observed compared to measurements at 45% RH. Additionally, KPFM operation does not seem to play a crucial role in this specific situation (see Supporting Information File 1, section SI.2).

**Figure 2:**
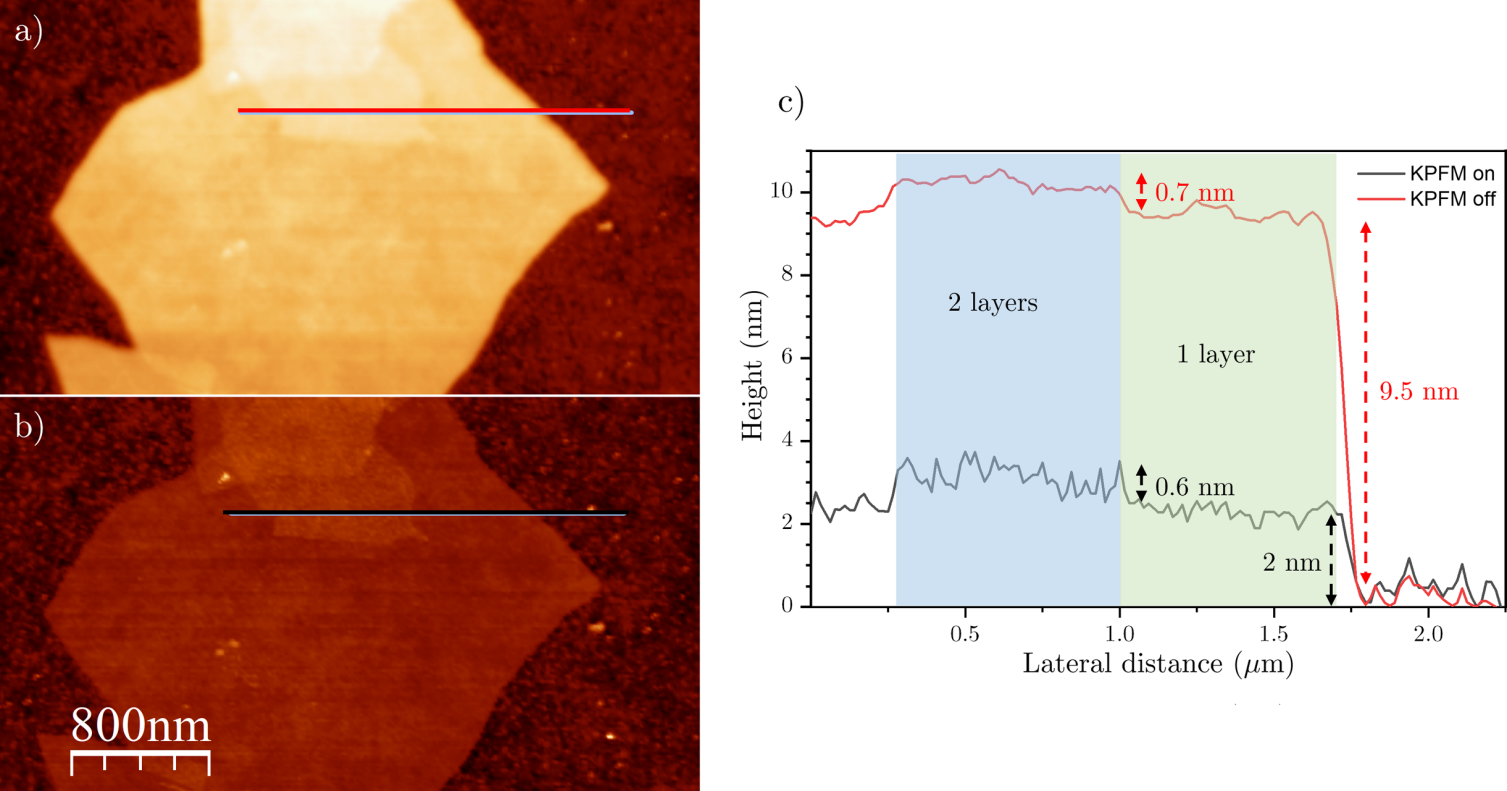
Topography images of charged rGO flakes. (a) KPFM off and (b) KPFM on, *z* scale = 15 nm. (c) Profiles along the red and black lines shown in (a) and (b).

Nevertheless, there are situations in which compensating the local surface potential through KPFM significantly enhances the precision of topography measurements, especially when the voltage between tip and sample is large. This is exemplified in Figure 2, where the tip–rGO voltage is intentionally modified by charging the flakes through bringing the tip into contact while applying an external bias voltage to the tip [78] (see Supporting Information File 1, section SI.3 for further details). Following the charging process, without activating the KPFM feedback (Figure 2a), there is a noticeable increase in the flake’s apparent height, reaching approximately 9 nm, while the typical height (about 2 nm) is restored only after turning on the KPFM loop (Figure 2b and Supporting Information File 1, section SI.3). This has significant implications in multilayer systems, where height is commonly used to determine the number of layers, and measurements without KPFM might give an erroneous layer count. Moreover, a line profile along one and two layers (Figure 2c) reveals that, after charging, the height increase of the first layer with respect to the substrate is much more pronounced than the increase of the second layer with respect to the first one, which remains essentially unchanged. Additional charging experiments (not shown) reveal that the measured flake height depends on the charging state; this effect is magnified as the amount of charge is increased, regardless of its sign. We could associate this behavior with the existence of an uncompensated electrostatic force that is nullified when KPFM is activated. However, it is crucial to remember that, under our measurement conditions, the tip always oscillates at resonance and amplitude variations are related to dissipative interactions. Since the electrostatic force is conservative, it should not change the oscillation amplitude [39]. Therefore, an increase in height correlates with larger dissipation, which prompts a tip retraction to maintain a constant amplitude. This suggests the presence of a voltage-dependent dissipation mechanism.

**Figure 3:**
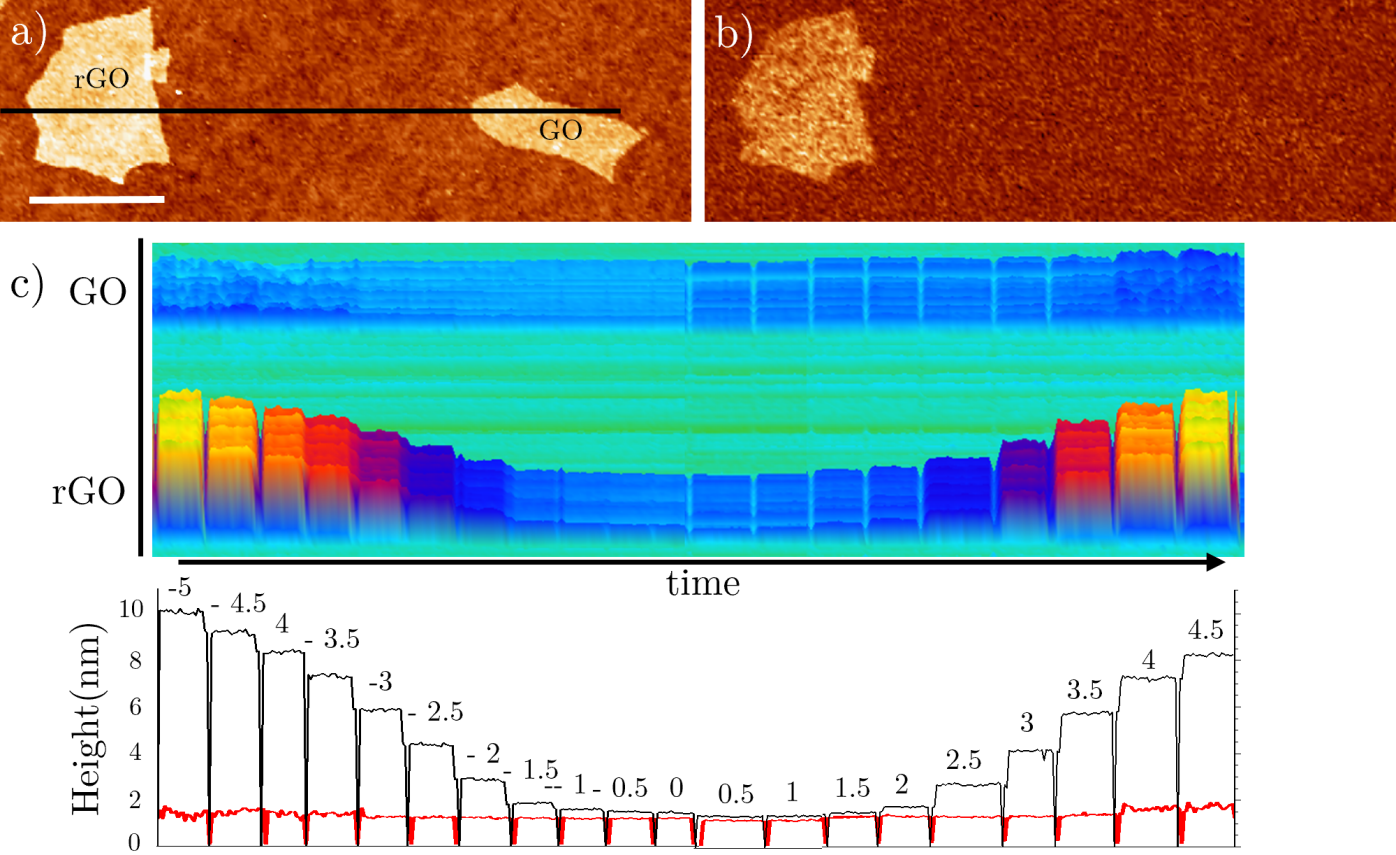
(a) Topography and (b) 2ω_elec_ images of rGO and GO monolayer flakes. (c) The top image corresponds to the 3D representation of the topography along the black line shown in (a) as a function of time as *V*_bias_ is changed in 0.5 V increments. The bottom plot corresponds to the average height of rGO (black) and GO (red) as a function of time for several discrete values of *V*_bias_. Each data point is calculated by averaging the height over each flake profile at a given time.

Since the apparent flake height seems to depend on both the tip–sample voltage and on the material, we explore these correlations on both GO and rGO flakes by biasing the tip with a DC voltage. To prevent any interaction between flakes arising from charge transfer through the substrate [79], we deliberately chose two well-separated rGO and GO single-layer flakes (Figure 3a,b). The AC voltage and the *y* scan are turned off at the highlighted profile in Figure 3a, and a DC voltage is applied to the tip (*V*_bias_) for a few seconds, which is then changed to another voltage in steps of 0.5 V. In Figure 3c, we display the variations in height as a function of *V*_bias_ along the line profile. In the case of GO, the height remains relatively constant with respect to the applied bias, showing only a slight increase at significantly positive and negative voltages (above ±4 V). In contrast, the rGO flake exhibits a much more pronounced dependence. At low bias voltages around the SP, the height is independent of *V*_bias_, but it rapidly increases at higher bias voltages. This confirms the presence of a voltage-dependent dissipation mechanism, particularly enhanced on rGO. However, it is important to emphasize that this height dependence with the voltage is not seen when the flakes are supported on a conducting substrate (see Supporting Information File 1, section SI.4).

**Figure 4:**
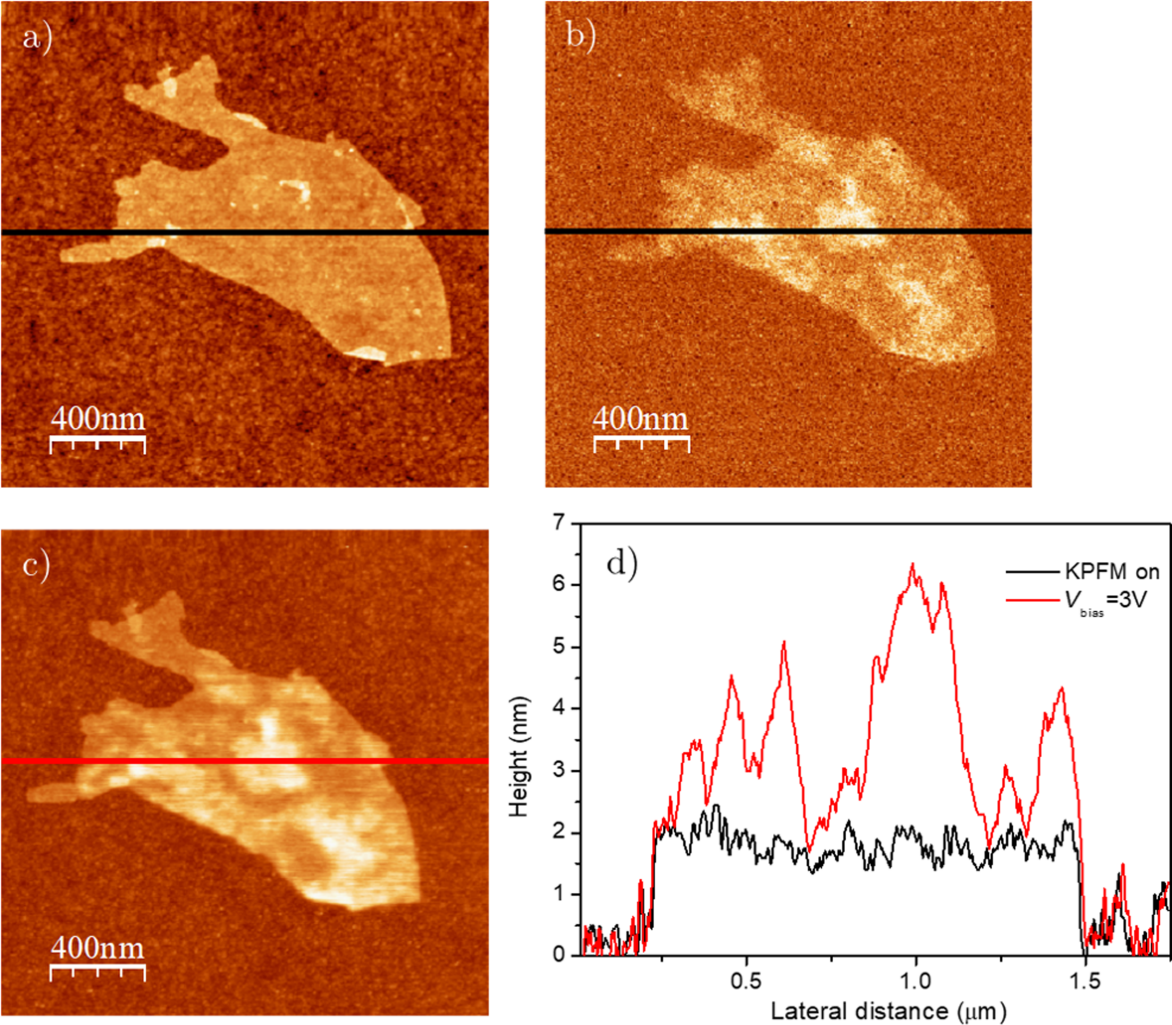
(a) Topography and (b) 2ω_elec_ images with KPFM on of a partially reduced rGO flake. (c) Topography of the same flake but with KPFM off and *V*_bias_ = 3 V. (d) Line profiles along the black and red lines in (a–c).

Moreover, we can examine a nanoscale heterogeneous sample, such as a partially reduced rGO flake. The transition from GO to rGO under reduction with hydrazine involves a non-homogeneous process, in which, at the intermediate reduction stages, the rGO flakes are composed of regions with different reduction degrees and, therefore, different properties [61]. As depicted in Figure 4a, when the tip–sample voltage is compensated (KPFM on), the height of rGO is roughly homogeneous along the flake. Conversely, when biasing the tip, not only does the overall height increase, but the increase is more pronounced in certain regions (Figure 4c,d). Comparing these regions with the 2ω_elec_ image (Figure 4b), we find that the height increase is correlated to the local degree of reduction, with a lateral size for the domains that varies from tens to hundreds of nanometers. This confirms, on the one hand, that this mechanism achieves nanoscale resolution, primarily attributable to the tip, and, on the other hand, that the voltage dissipation mechanism depends on the local properties of the material.

**Figure 5:**
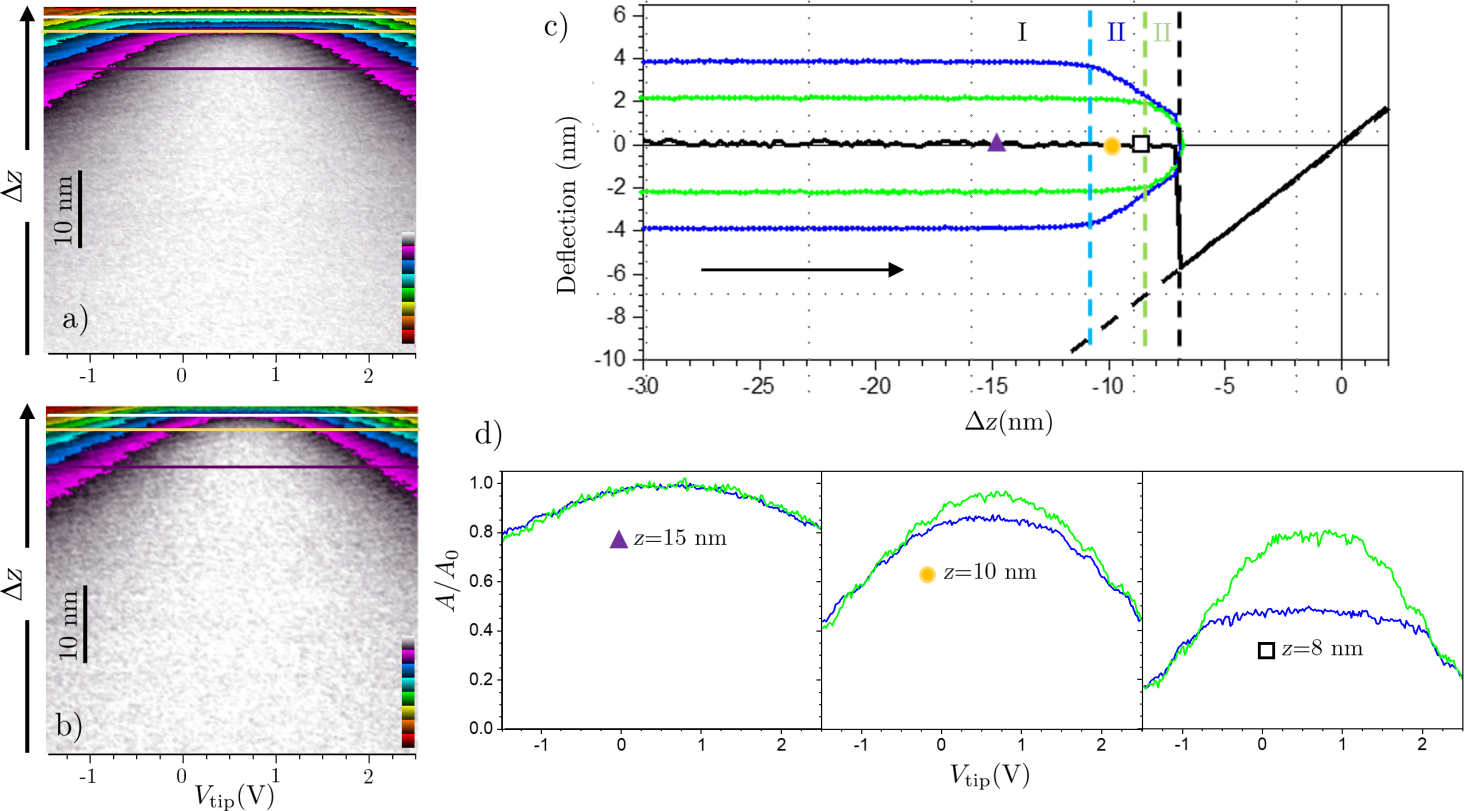
A(*V*_bias_, *z*) 3D spectroscopy images acquired on a rGO monolayer flake for (a) *A*_0_ = 4 nm and (b) *A*_0_ = 2 nm. Only the non-contact region is shown. (c) Deflection vs mean distance curve (black). The green (*A*_0_ = 2 nm) and blue (*A*_0_ = 4 nm) curves represent the upper and lower turning points of the oscillating tip at *V*_bias_ = SP. The corresponding green and blue dashed lines mark the boundary between regions I and II for the amplitudes, while the black dashed line indicates the jump to the contact point. (d) *A*/*A*_0_ vs *V*_bias_ for different tip–sample distances; the green and blue curves correspond to *A*_0_ = 2 nm and *A*_0_ = 4 nm, respectively.

To gain a deeper understanding of this voltage-dependent dissipative interaction, we conducted 3D spectroscopy measurements on rGO. The amplitude vs *V*_bias_ as a function of tip–sample distance images (*A*(*V*_bias_, *z*)) for two different free oscillation amplitudes (*A*_0_) are shown in Figure 5a (*A*_0_ = 4 nm) and Figure 5b (*A*_0_ = 2 nm). From the corresponding normal force and frequency shift channels (not shown) and following the data processing described in [63], we derived the deflection–distance curve, as well as the SP for each tip–sample distance, SP(*z*). This approach enabled us to accurately calibrate the tip–sample distance and the oscillation amplitude as shown in Figure 5c, where, in addition to the deflection–distance curve, we also include the upper and lower turning points of the oscillating tip at the minimum tip–sample voltage *A*(*V*_bias_ = SP, *z*), where *z* is the mean tip–sample distance around which the tip is oscillating. In Figure 5c, we identify the two well-known non-contact interaction regimes, namely, (I) distances far from the sample, where capillary forces are negligible, and (II) distances close to sample, where the formation and rupture of liquid necks starts, and the amplitude reduction due to capillary forces becomes relevant [35,37]. The boundary between these regimes, of course, depends on *A*_0_ since, at the same average tip–sample distance, for larger amplitudes, the lower turning point is closer to the sample, favoring the earlier formation of liquid necks.

In Figure 5d, representative amplitude vs *V*_bias_ curves at different tip–sample distances are displayed. To facilitate comparison between various oscillation amplitudes, we present the amplitude referred to its free amplitude in each case (*A*/*A*_0_). At large distances (*z* = 15 nm), the amplitude exhibits a clear parabolic dependence on the bias voltage, being equal to the free oscillation (*A*/*A*_0_ = 1) at *V*_bias_ = SP and lower than the free oscillation (*A*/*A*_0_ < 1) when *V*_bias_ ≠ SP. This confirms the presence of a dissipation mechanism depending on the tip–sample voltage even at large tip–sample distances. As the tip approaches (*z* = 10 nm), the purely parabolic dependence is retained only for the lower oscillation amplitude. Conversely, a nearly voltage-independent flat region around the SP is superimposed on the curve with the larger oscillation amplitude. This is explained by considering that, at this mean tip–sample distance, we are still in regime I for the low amplitude while transitioning to regime II for the larger amplitude. In regime II, dissipation due to liquid necks dominates at low tip–sample bias voltages [35], while the voltage-dependent dissipative term becomes prominent at higher *V*_bias_. Finally, at *z* = 8 nm, where both amplitudes are in regime II, the curves exhibit a combination of voltage-independent dissipation at *V*_bias_ around the SP and a voltage-dependent dissipative contribution at large tip–sample voltages. The relative contribution of each term to the total dissipation depends on the free oscillation amplitude. In contrast to the voltage-independent contribution mediated by capillary forces [38,63], the voltage-dependent dissipation contribution perfectly scales with the free oscillation amplitude. This scaling suggests that, for low to moderate amplitudes, this dissipation mechanism is proportional to *A*_0_. The same experiments were conducted on the GO flake (see Supporting Information File 1, section SI.5), revealing that, in this material, the voltage-dependent contribution is significantly lower, aligning well with the observations of Figure 3.

In summary, our experimental results confirm that, in addition to the well-known dissipative interaction due to capillary forces, in 2D materials, there is another dissipative interaction, whose magnitude is tightly related to the tip–sample voltage and strongly depends on the 2D material itself. Moreover, this mechanism achieves nanoscale resolution and is sensitive to local heterogeneities in the material’s properties. We also show that this effect is particularly relevant whenever the 2D material is supported on an insulating substrate and can be an important source of error when determining its thickness.

According to its voltage dependence, it is reasonable to assume an electronic origin. Previously, it has been proposed in other systems that a plausible explanation to this effect is related to Joule dissipation [80–81]. The physical processes that lead to Joule dissipation are as follows: (i) In the presence of an effective tip–sample voltage, the oscillating AFM tip generates a time-dependent electrical field, which induces a current and Joule dissipation due to the non-zero resistance of the material. These currents induce an oscillating charge movement. (ii) Simultaneously, these moving charges also generate a time-dependent electric field, which needs to be considered consistently. We think that our findings align well with this explanation, as a key difference between GO and rGO is their electrical conductivity. Moreover, the conduction mechanism in GO and rGO flakes is variable-range hopping [75], and slight changes in the reduction degree may vary the conductivity by many orders of magnitude [69], which would explain the high sensitivity of the measurements to the local reduction degree within one partially reduced rGO flake.

To account for Joule dissipation, different models have been derived with different approximations, most of them treating the metal–metal situation or modeling the tip–sample system as a series of capacitors [80–84]. In this work, we follow a different approach. We derive the general equations in the macroscopic limit for the charge dynamics near the surface between two media. In a previous work, we studied the tip-less case, which we will use as the starting point for our model. Here, we just summarize the main aspects and refer to [78] for further details. Then, we include the tip influence by adding the appropriate boundary condition, which is time-dependent when the tip is oscillating and/or an AC voltage is applied. After adding this contribution, we particularize to 2D materials on insulating supports, where the current through the bulk is minimal and the induced currents are confined to the surface.

**Figure 6:**
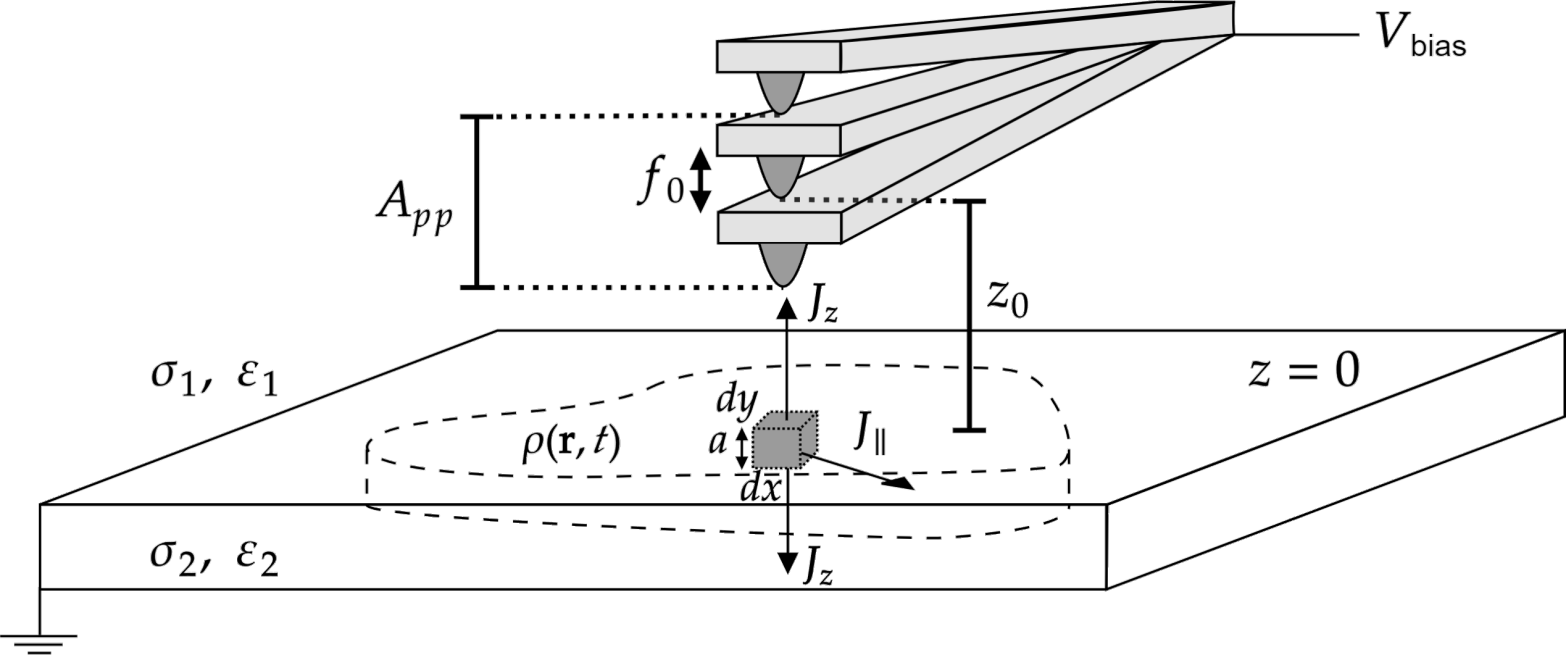
Scheme of the modelled system with an oscillating tip above the sample’s surface. The tip is oscillating at its resonant frequency, *f*_0_, with a peak-to-peak amplitude *A*_pp_. The current density (**J**(*x*, *y*, *z*, *t*)) leaving the differential volume element (d*V* = d*x*d*y a*) has components parallel (*J*_∥_ = σ_∥_*E*_∥_) and perpendicular (*J_i,z_* = σ*_i_E_i,z_*, with *i* = 1,2 above and below the surface, respectively) to the surface.

As shown in Figure 6, the sample is modelled as a semi-infinite medium with permittivity ε_2_ and bulk conductivity σ_2_ embedded in a medium with permittivity ε_1_ and conductivity σ_1_. A well-known key feature is that, for an homogeneous bulk system, an arbitrary initial charge density, ρ(**r**, *t* = 0), decays in time as ρ(**r**, *t*) = ρ(**r**, *t* = 0)·exp(−*t*/τ). This result is directly derived from the Poisson equation (∇·**E** = ρ/ε), the continuity equation (∇·**J** = −∂ρ/∂*t*), and Ohm’s law (**J** = σ**E**). According to this result, we assume that bulk regions not initially charged will remain uncharged, that is, all charge (if any) will be located at the surface. In order to avoid possible singularities, we assume that all non-negligible charge density is located in a region of height *a* inside the sample (from *z* = 0 + ε→0^+^ to *z* = *−a*, see Figure 6). We note that, as long as *a* is finite, we must handle three-dimensional quantities (charge density and conductivity). Only when we take the limit *a*→0, two-dimensional quantities will become properly defined. We assume that for currents parallel to the surface inside the gray region, *J*_∥_ = σ_∥_*E*_∥_, the conductivity is different to the bulk value. Then, the final surface conductivity will be defined as σ_s_ = lim*_a_*_→0_*a*σ_||_. Similarly, the two-dimensional charge density is defined as *n*(*x*, *y*, *t*) = lim*_a_*_→0_*a*ρ(*x*, *y*, *z* = 0, *t*). Now, applying charge conservation (continuity equation), Ohm’s law, and Gauss’ theorem to the gray surface element in the sample plotted in Figure 6 leads to [78]:


[1]

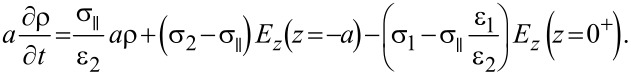



*E_z_*(*x*, *y*, *z*, *t*) is the *z* component of the electric field. The electric field is induced by both the presence of the tip (at voltage *V*_bias_) and by the surface charge density. The electric field must then verify Maxwell’s first equation, ∇·**E** = ρ/ε, above and below the surface, whose general solution is


[2] $$\begin{array}{c} V\left(x,y,z,t\right)=V_{\text{tip}}\left(x,y,z,t\right)\\ +\int \text{d}x^{\prime }\int \text{d}y^{\prime }\;n\left(x^{\prime },y^{\prime },t\right)V_{1}\left(x^{\prime },y^{\prime },z^{\prime };x,y,z,t\right), \end{array}$$

where *V*_tip_(*x*, *y*, *z* , *t*) is the potential generated by the tip in the absence of charges, the second term on the right-hand side is the potential generated by the charges at the surface, *n*(*x*, *y*, *t*) is the surface charge density, and *V*_1_(*x′*, *y′*, *z′*; *x*, *y*, *z*, *t*) is the potential at position (*x*, *y*, *z*) generated by a unit charge located at (*x′*, *y′*, *z′*). *V*_tip_(*x*, *y*, *z*, *t*) is calculated through solving the Laplace equation with the boundary condition *V*(**r**∈ tip surface) = *V*_bias_. This term may depend on time if the tip oscillates or if an AC voltage is applied. *V*_1_(*x′*, *y′*, *z′*; *x*, *y*, *z*, *t*) is calculated with the boundary condition *V*(**r**∈ tip surface) = 0 and is proportional to the standard Green function of the problem (in some textbooks *G*(**r**, **r**′) is the potential at **r** due to a charge of value *q* = −4πε located at **r**′ [85]).

Equation 1 and Equation 2, together with initial and boundary conditions constitute a closed set to determine *n*(*x*, *y*, *t*). It is possible to differentiate Equation 2 to obtain a similar equation for *E_z_*(**r**). Then, inserting it into Equation 1 and taking the limit *a*→0, we obtain an integro-differential equation for *n*(*x*, *y*, *t*) that defines the exact macroscopic limit of the problem of charge dynamics at the surface including the effect of the tip. The complexity of this equation impedes an analytical treatment unless we introduce certain approximations that enable us to analyze it and deduce some of its consequences. In our case, (i) the tip is described as a sphere of radius *R* located at (*x* = 0, *y* = 0, *z* = *z*_0_), and (ii) we take the long-distance limit, where the tip is far away from the surface; thus, we only keep the leading-order terms in *z*_0_→∞. This approximation allows for a simple treatment in terms of image charges, see for example [86–87]. In general, verifying the boundary conditions at the surface and at the tip surface requires to treat an infinite series of image charges, but for large *z*_0_ these charges become rapidly negligible. Taking this into account, the term *V*_tip_(*x*, *y*, *z* ,*t*) can be written as


[3] $$V_{\text{tip}}\text{​}\left(x,y,z,t\right)\;\approx \;\{\begin{array}{ll} \frac{q}{4\pi \varepsilon_{1}}\left(\begin{array}{l} \frac{1}{\sqrt{x^{2}\;+\;y^{2}\;+\;{\left(z\;-\;z_{0}\right)}^{2}}}\\ -\frac{s}{\sqrt{x^{2}\;+\;y^{2}\;+\;{\left(z\;+\;z_{0}\right)}^{2}}} \end{array}\right) & \text{for }z>0,\\ \frac{q}{4\pi \varepsilon_{2}}\frac{\left(1+s\right)}{\sqrt{x^{2}+y^{2}+{\left(z-z_{0}\right)}^{2}}} & \text{for }z<0, \end{array}$$

where *s* = (ε_2_ − ε_1_)/(ε_2_ + ε_1_), and *q* = 4πε_1_*RV*_bias_. *V*_bias_ and *z*_0_ may be time-dependent if the tip is oscillating or a variable voltage is applied. Similarly, the potential generated by a unit charge, *V*_1_(*x′*, *y′*, *z′*; *x*, *y*, *z*), for *z*, *z′* < 0, is written as


[4] $$\begin{array}{c} V_{1}\text{​}\left(x^{\prime },y^{\prime },z^{\prime };x,y,z\right)\;\approx \frac{1}{4\pi \varepsilon_{2}\sqrt{{\left(x\;-\;x^{\prime }\right)}^{2}\;+\;{\left(y\;-\;y^{\prime }\right)}^{2}\;+\;{\left(z\;-\;z^{\prime }\right)}^{2}}}\\ +\frac{s}{4\pi \varepsilon_{2}\sqrt{{\left(x\;-\;x^{\prime }\right)}^{2}\;+\;{\left(y\;-\;y^{\prime }\right)}^{2}\;+\;{\left(z\;-\;z^{\prime }\right)}^{2}}}. \end{array}$$

We note that, once *E_z_* is calculated and substituted into Equation 1, the right-hand side of the integro-differential equation contains two kind of terms, namely, (i) terms independent of *n*(*x*, *y*, *t*) and (ii) terms linear in *n*(*x*, *y*, *t*). The interpretation of the first kind of terms is straightforward. In the absence of charges, the tip produces electric fields and, thus, currents. These currents modify the charge density through the continuity equation. Collecting all the terms of the first kind and taking the limit *a→ 0* leads to


[5] $$\begin{array}{c} f_{\text{tip}}\left(x,y\right)=\frac{2Rz_{0}V_{\text{bias}}}{\varepsilon_{1}+\varepsilon_{2}}\frac{\left(\varepsilon_{2}\sigma_{1}-\varepsilon_{1}\sigma_{2}\right)}{{\left(x^{2}+y^{2}+z_{0}^{2}\right)}^{3/2}}\\ +\frac{2\varepsilon_{1}V_{\text{bias}}R\sigma_{s}}{\varepsilon_{1}+\varepsilon_{2}}\frac{\left(x^{2}+y^{2}-2z_{0}^{2}\right)}{{\left(x^{2}+y^{2}+z_{0}^{2}\right)}^{5/2}}. \end{array}$$

Furthermore, within our approximations, *V*_1_ is not affected by the tip, so it can be written as *V*_1_(*x* − *x′*, *y* − *y′, z*, *z′*). Then, the second kind of terms in Equation 1 become a pure convolution and can be easily Fourier-transformed in the plane (see Appendices A and B in [78] for details). After Fourier transformation and taking the limit *a*→0, Equation 1 becomes [78]:


[6] $$\frac{\partial n\left(k,t\right)}{\partial t}=\frac{-n\left(k,t\right)}{\tau \left(k\right)}+f_{\text{tip}}\left(k\right),$$

where 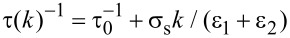, τ_0_ = (ε_1_ + ε_2_)/(σ_1_ + σ_2_), and




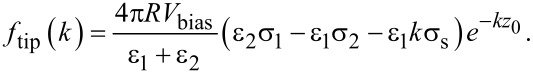




It should be noted that Equation 6 only depends on *k* = |**k**| since radial symmetry is assumed. For a non-oscillating tip (*z*_0_ and *V* constant), this equation can be solved analytically in Fourier space. A first consequence in this case is that, as *t*→∞, there will be a surface charge density that will depend on the bulk conductivities and the surface conductivity,


[7] $$\begin{array}{c} n\left(k,t\to \infty \right)=f_{\text{tip}}\left(k\right)\tau \left(k\right)\\ =4\pi RV_{\text{bias}}\frac{\varepsilon_{2}\sigma_{1}-\varepsilon_{1}\sigma_{2}-\sigma_{\text{s}}\varepsilon_{1}k}{\sigma_{1}+\sigma_{2}+k\sigma_{\text{s}}}e^{-kz_{0}}. \end{array}$$

This equation shows that, initially, the tip generates an electric field that gives rise to currents at the surface, charging it in the process. This process stops when there is no net electric field parallel to the surface, and thus the whole surface is set at a constant voltage. The surface charge distribution is such that it cancels the electric field parallel to the surface, *E*_∥_, generated by the tip. The final voltage depends on the geometry of the problem and the conductivity of each media. Solutions of this kind will exist regardless of the model used for the tip.

In our model, a 2D material supported on an insulating substrate is described by setting σ_1_, σ_2_ ≈ 0, that is, bulk conduction is negligible, but surface conductivity is not. Then, for an initially uncharged surface (*n*(*r*, *t* = 0) = 0), we get


[8] $$n\left(r,t\right)=-8\pi^{2}\varepsilon_{1}RV_{\text{bias}}\cdot \left(\frac{z_{0}}{{\left(r^{2}+z_{0}^{2}\right)}^{3/2}}-\frac{{\left(\varepsilon_{1}+\varepsilon_{2}\right)}^{2}}{{\left[\sigma_{s}t+\left(\varepsilon_{1}+\varepsilon_{2}\right)z_{0}\right]}^{2}{\left(1+\frac{{\left(\varepsilon_{1}+\varepsilon_{2}\right)}^{2}r^{2}}{{\left[\sigma_{s}t+\left(\varepsilon_{1}+\varepsilon_{2}\right)z_{0}\right]}^{2}}\right)}^{3/2}}\right),$$

where 
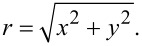
 This solution shows that there is a relatively slow charging of the surface with a characteristic time


[9] $$\tau \left(k=1/z_{0}\right)\approx \frac{\left(\varepsilon_{1}+\varepsilon_{2}\right)z_{0}}{\sigma_{s}},$$

such that for *t* ≪ τ, the surface remains uncharged, but for times larger than τ, the surface will be charged, even if no charges were present at *t* = 0. When τ is much larger than any relevant time, this charge will be negligible, while as τ→0, the surface charge density will correspond to the metallic case. In our samples, the local surface conductivity varies by many orders of magnitude and, thus, this effect may influence the measurements. A rough estimation for GO and highly reduced GO gives an idea of typical timescales for an insulating and a conducting 2D material. At a typical working distance, *z*_0_ ≈ 10 nm, and using the σ_∥_ values from [69] (σ_∥_^GO^ ≈ 10^−8^ S/m and σ_∥_^rGO^ ≈ 10^5^ S/m), the surface conductivity is calculated as σ_s_ = σ_∥_*a*, with *a* ≈ 1 nm, the thickness of the flake. We also consider ε_1_ = ε_0_ and 
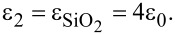
 This yields relaxation times that span many orders of magnitude, from tens of milliseconds to few femtoseconds (τ_GO_ ≈ 40 ms and τ_rGO_ ≈ 4 fs). Comparing these with typical acquisition times for a data point, *t*_p_ ≈ 10^−3^ s, yields two opposite situations. For GO, *t*_p_ < τ_GO_, the surface charge distribution does not reach the equilibrium configuration during the acquisition time, while for rGO, *t*_p_ ≫ τ_rGO_, measurements are carried out in equilibrium. It is important to note that the time evolution in Equation 8 is entirely a consequence of a non-negligible σ_s_; if we set σ_s_ = 0, the time evolution cancels, and we recover the expected solution for a dielectric interface.

When the tip oscillates or an AC voltage is applied, the oscillating electric field produces Joule dissipation at the surface as well as an oscillating surface charge. For simplicity, we consider the case of an AC voltage (*V*_bias_ = *V*_DC_ + *V*_AC_cos(ω*t*)), although solutions will be very similar for small tip oscillation amplitudes, since only *f*_tip_(*k*) depends on *z*_0_ or *V*. In order to properly calculate them, we need to solve Equation 1 including the oscillating term *V*_AC_cos(ω*t*). As this change only affects *f*_tip_(*k*), we can find the oscillating part of the solution for each *k* applying the variation of constants method, thus:


[10] $$n_{\text{AC}}\left(k,t\right)=\frac{V_{\text{AC}}f_{\text{tip}}\left(k\right)}{1+\tau^{2}\left(k\right)\omega^{2}}\left[\cos\left(\omega t\right)+\tau \left(k\right)\omega \sin\left(\omega t\right)\right].$$

We note that this oscillating charge must be taken into account in order to properly calculate the tip–sample interaction. To our knowledge, the effect of this time-dependent charge distribution has not been taken into account, and it could be important when using KPFM, specially when τ^−1^ is comparable to the oscillation frequency and when the AC voltage is large.

Now *V*(*k*, *z*, *t*) can be calculated substituting *n*_AC_(*k*, *t*) into the Fourier transform of Equation 2. From *V*(*k*, *z*, *t*), it is possible to calculate the Joule dissipation. In particular, for our case (σ_1_, σ_2_ = 0), the dissipated power, *P*, takes the simple expression:


[11] $$P=\sigma_{\text{s}}\frac{\omega }{2\pi }\int_{0}^{2\pi /\omega }\text{d}t\int \text{d}k\;k^{2}{\left|V\left(k,z=0,t\right)\right|}^{2}.$$

Because of the linearity of Equation 2, the oscillating part of the total electrostatic potential at the surface will be linear in *V*_AC_. Hence, this equation predicts the parabolic dependence of the dissipation with the applied bias voltage and shows that, in 2D materials, there is an a priori non-negligible dissipation mechanism related to the in-plane conductivity. This might also explain why the application of KPFM mostly mitigates the issue of incorrect height measurements in 2D materials, although it does not entirely correct the problem. Through the use of KPFM, the overall tip–sample voltage across a specific region beneath the tip is nullified, minimizing the mean electric field in that area and, consequently, effectively reducing the currents. However, it is important to note that, locally, complete nullification of the electric field may not occur, resulting in residual small surface currents with an associated Joule dissipation.

## Conclusion

We conducted a study on a system involving GO and rGO flakes supported on SiO_2_/Si to elucidate the origin behind consistently inaccurate AM-AFM height measurements of 2D materials when they are deposited on insulating substrates. Our experimental results reveal that this discrepancy arises from a dissipation mechanism directly proportional to the free oscillation amplitude for low to moderate amplitudes. Additionally, we observed a parabolic dependence on the tip–sample bias voltage, with the dissipation effect being more pronounced as the in-plane conductivity of the material increases.

We have linked this phenomenon to an in-plane Joule dissipation due to an oscillating charge induced by the oscillating tip, which is enhanced when the underlying substrate is insulating. To comprehensively understand this 2D Joule dissipation, we have established the equations governing surface charge dynamics, accounting for the presence of the oscillating tip. While solving the exact equations proves challenging without resorting to numerical techniques, we have derived a comprehensive explanation of the experimental observations by considering the long-distance limit. Moreover, our model reveals that, aside from the impact of Joule dissipation on height measurements in AM-AFM mode, the presence of an oscillating surface charge introduces additional dynamic effects typically overlooked, which, under certain conditions, should be considered when calculating the tip–sample interaction. Also, we have identified a relaxation time required to reach the stationary or periodic state for a static or oscillating tip, respectively. This relaxation time is crucial to the overall charge dynamics and depends on the material’s in-plane conductivity and on the measurement parameters. When this relaxation time is comparable to, or even significantly exceeds, the characteristic AFM measurement times, it may influence not only the topography, but also other interaction channels, particularly, those related to electrostatic properties.

Finally, it is worth noting that the oscillating surface charge on the material’s surface may not only be induced by the scanning tip but also by applying an external AC voltage. This approach presents distinct advantages, allowing for precise tuning of both amplitude and frequency, providing an alternative path for exploring the electronic properties of 2D materials, which will be addressed in the future.

## Supporting Information

Supporting information includes: SI.1. Structure of GO and rGO. SI.2. Experiments at different relative humidity. SI.3. Charging of rGO flakes. SI.4. rGO on a conducting substrate. SI.5. 3D spectroscopy on a GO monolayer flake.

File 1 Additional data.

## Data Availability

The data that supports the findings of this study is available from the corresponding author upon reasonable request.
