# Supplementary material for: Exploring surface charge dynamics: implications for AFM height measurements in 2D materials
Source: Beilstein J Nanotechnol. 2024 Jul 1;15:767–80. doi: 10.3762/bjnano.15.64 (PMC11228822; doi:10.3762/bjnano.15.64)
Supplement: File 1 — Additional data. [file Beilstein_J_Nanotechnol-15-767-s001.pdf]

BEILSTEIN JOURNAL OF NANOTECHNOLOGY

Supporting Information

for

Exploring surface charge dynamics: implications for AFM height measurements in 2D materials

Mario Navarro-Rodriguez, Andres M. Somoza and Elisa Palacios-Lidon

Beilstein J. Nanotechnol. **2024,** *15,* 767–780. doi:10.3762/bjnano.15.64

Additional data

License and Terms: This is a supporting information file under the terms of the Creative Commons Attribution License (https://creativecommons.org/licenses/by/4.0). Please note that the reuse, redistribution and reproduction in particular requires that the author(s) and source are credited and that individual graphics may be subject to special legal provisions.
The license is subject to the *Beilstein Journal of Nanotechnology* terms and conditions: (https://www.beilstein-journals.org/bjnano/terms)

SI.1 Structure and properties of GO and rGO

Graphene oxide (GO) is a highly disordered and non-stoichiometrical 2D material, which belongs to the family of graphene-related materials. As shown in Figure S1a, the honeycomb structure of graphene is distorted by the addition of numerous defects and oxygen-containing functional groups such as carboxyl, epoxy, and hydroxy groups [1]. These groups change the hybridization from sp^2 to sp^3 [2]. This disrupts the planar structure and affects the conduction properties of the material, making it highly insulating. Specifically, GO typically exhibits a C/O ratio between 1.5 and 2.5 [1], and an sp^2/sp^3 ratio ranging from 0.4 to 0.6 [3], highlighting its disordered nature. This disorder leads to Efros–Shklovskii variable-range hopping (ES-VRH) conduction, even at room temperature [4]. The removal of these oxygen-containing functional groups (through any method) is known as reduction of GO and results in rGO. As shown in Figure S1b, in contrast to GO, the amount of oxygen containing functional groups is reduced; however, the material remains disordered and non-stoichiometric. For rGO, the C/O ratio is typically greater than 8 [1], and the sp^2/sp^3 ratio is above ≈ 0.6. As the flakes are reduced, localized domains with restored sp^2 hybridization appear over the flake, which in turn increase in the localization length of the electrons in the material. As a consequence, during the reduction process, the conductivity increases by several orders of magnitude as the size of these domains increases; thus, the resulting conductivity depends on the amount of oxygen-containing functional groups removed. Nevertheless, it should be noted that the graphene structure is never fully recovered as some functional groups and defects remain after reduction.

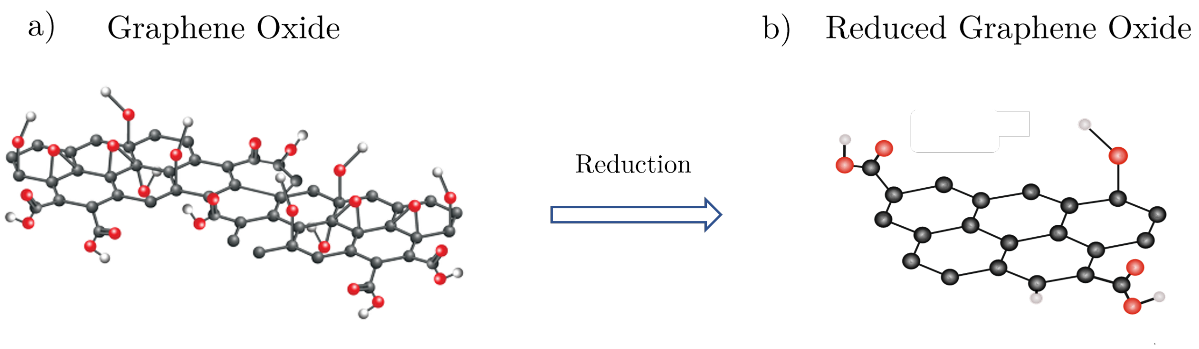

Figure S1: Schematic depiction of the structure of (a) GO and (b) rGO.

SI.2 Experiments at different relative humidity

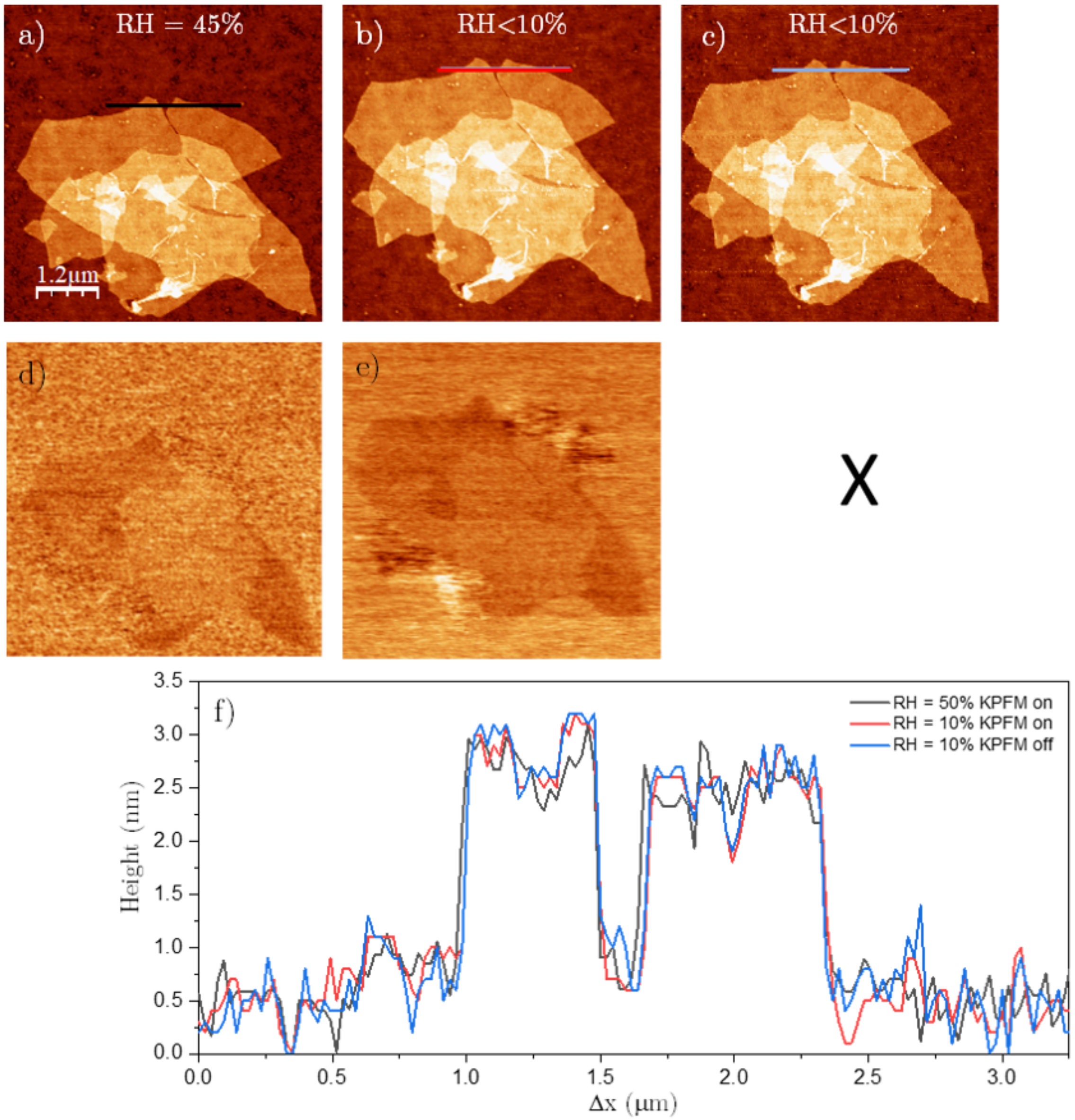

Figure S2: Top panel: topography images at (a) RH= 45% and (b, c) RH < 10%. The z scale in all images is 7 nm. (d, e) KPFM images (z = 350 mV) of (a) and (b), respectively. (f) Line profiles marked in (a–c).

SI.3 Charging of rGO flakes

In Figure S3, we show the effect of compensating the surface potential (SP) for two different situations. In Figure S3a, we show the topography of a group of rGO flakes with KPFM on (Figure S3b) before charging. In this situation, turning KPFM off yields no difference on the apparent height (Figure S3c). After measuring the topography with both KPFM on and off, we establish mechanical contact between tip and sample and apply a bias pulse while in contact, charging the flake in the process, as shown on Figure S3d. After this charging procedure, we measure the same area again with KPFM off (Figure S3e), showing that the apparent height of the flake has increased from about 2 nm to about 10 nm, as detailed in the main text. To conclude the experiment, we turn on the KPFM loop and find that the apparent height coincides exactly with that of the uncharged flake (Figure S3f), and that the SP over the flake has increased considerably (Figure S3g).

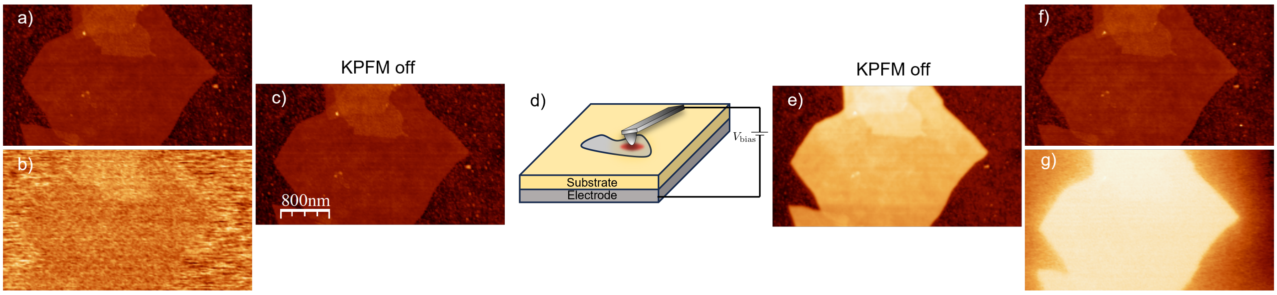

Figure S3: (a)Topography and (b) KPFM (z = 500 mV) images of uncharged rGO flakes. (c) Topography of the same uncharged flakes with the KPFM loop off. (d) Drawing of the charging procedure. (e) Topography of the flakes after charging with KPFM off. (f) Topography and (g) KPFM (z = 4.5 V) images of charged rGO flakes.

SI4. rGO on a conducting substrate

In Figure S4, we present two panels corresponding to two different experiments performed on rGO deposited on HOPG. In the top panel, we study the effect of changing V_{bias} while scanning on an apparent rGO flake. Figure S4a shows to the topography of a monolayer rGO flake. The corresponding KPFM image, Figure S4b, allows one to better discern the shape and geometry of the flake, showing that its bottom-right edge is folded onto itself and has a slightly different SP compared with the rest of it. After taking these images, we measure same area again; but this time, we stop the y scan at the thick black line in Figure S4c to study the evolution of an individual profile. At each of the thinner lines in Figure S4c, V_{bias} is changed to the value shown on the left of Figure S4c, and we find that the apparent height of the flake is not affected by the bias applied to the tip. The bottom panel of Figure S4 corresponds to a charge injection experiment performed on the same rGO flake. Figure S4d shows the topography of the rGO flake during a charge injection experiment. The charge injection is performed at the black line in Figure S4d according to the procedure detailed in the previous section. The KPFM image is simultaneously recorded (Figure S4e), and the KPFM loop is turned off just during the charge injection process and turned on again shortly after. In this experiment, we find that the SP is not changed by the charge injection. Since the flake is electrically connected to the substrate, all the injected charge quickly flows to the bulk after charging; thus, since the flake cannot be charged, the apparent height is not affected.

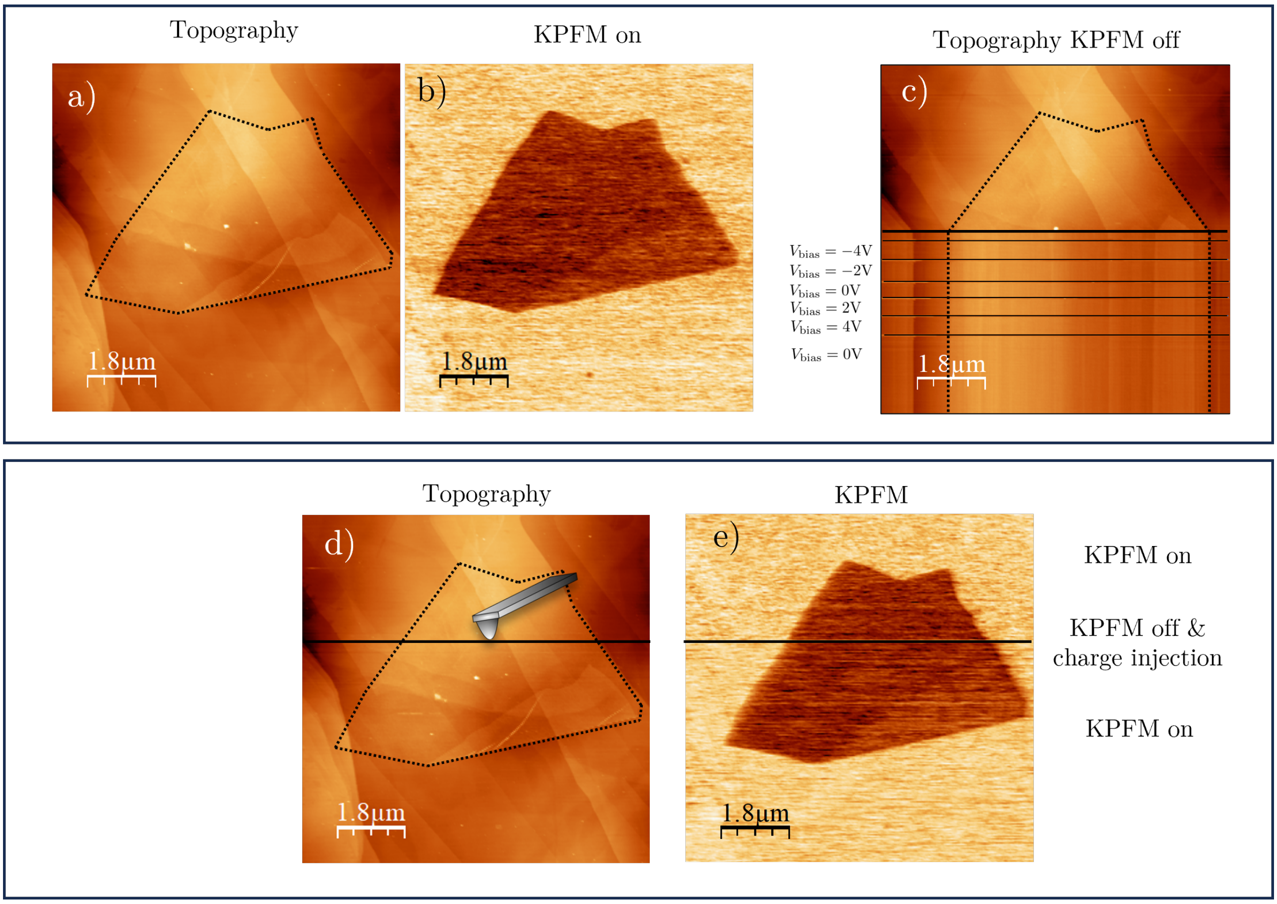

Figure S4: (a) Topography of an rGO flake deposited on HOPG. (b) KPFM image corresponding to the topography in (a). (c) Topography of the same rGO flake. The y scan is turned off at the thicker black line. V_{bias} is changed at each of the thinner black lines to the value specified on the left. (d) Topography image of an rGO flake during a charge injection experiment. The black line denotes the line at which the charge is injected. (e) KPFM image corresponding to the topography in (d). The KPFM loop is turned off during the charge injection and turned on just after finishing. z = 15 nm for all the topography images. z = 410 mV for all the KPFM images.

SI5. 3D spectroscopy on a GO monolayer flake

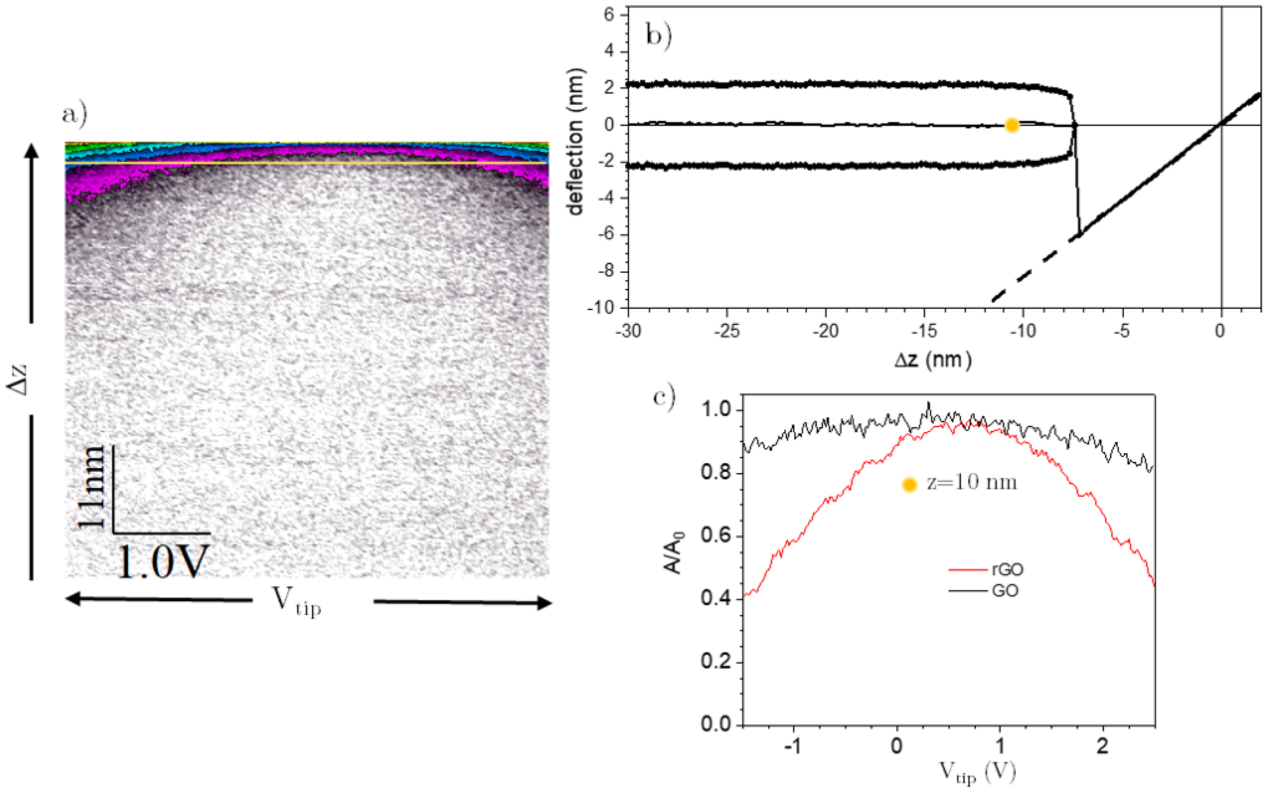

Figure S5: (a) $A(V_{tip}, z)$ image. (b, c) A/A_0 curves obtained on GO and rGO (black and red lines, respectively), at an average tip–sample distance z = 10 nm and A_0 = 2 nm.

References

1. Dimiev, A. M.; Eigler, S. *Graphene oxide: fundamentals and applications*; John Wiley & Sons, 2016; doi:10.1002/9781119069447.

2. Krishnamoorthy, K.; Veerapandian, M.; Yun, K.; Kim, S.-J. *Carbon* **2013**, *53*, 38–49. doi:10.1016/j.carbon.2012.10.013.

3. Mattevi, C.; Eda, G.; Agnoli, S.; Miller, S.; Mkhoyan, K. A.; Celik, O.; Mastrogiovanni, D.; Granozzi, G.; Garfunkel, E.; Chhowalla, M. *Adv. Funct. Mater.* **2009**, *19*, 2577–2583. doi:10.1002/adfm.200900166.

4. Palacios-Lidón, E.; Colchero, J.; Ortuno, M.; Colom, E.; Benito, A. M.; Maser, W. K.; Somoza, A. M. *ACS Mater. Lett.* **2021**, *3*, 1826–1831. doi:10.1021/acsmaterialslett.1c00550.
